# Effect of vitamin D_3_ and a stinging nettle extract on the gastric tissue of rats administered with trinitrobenzene sulfonic acid

**DOI:** 10.17221/111/2023-VETMED

**Published:** 2024-03-28

**Authors:** Arzu Gezer, Sukran Yediel Aras, Nurcan Kilic Baygutalp, Ebru Karadag Sari, Gursel Bedir, Behzad Mokhtare, Kadriye Yilmaz

**Affiliations:** ^1^Vocational School of Health Services, Ataturk University, Erzurum, Turkiye; ^2^Department of Midwifery, Faculty of Health Sciences, Kafkas University, Kars, Turkiye; ^3^Department of Biochemistry, Faculty of Pharmacy, Ataturk University, Erzurum, Turkiye; ^4^Department of Histology and Embryology, Faculty of Veterinary Medicine, Kafkas University, Kars, Turkiye; ^5^Department of Histology and Embryology, Ataturk University School of Medicine, Erzurum, Turkiye; ^6^Department of Pathology, Faculty of Veterinary Medicine, Dicle University, Diyarbakir, Turkiye

**Keywords:** aquaporin-1, Crohn’s disease, somatostatin, TNF-α

## Abstract

In this study, the effects of vitamin D_3_ (Vit. D) and a stinging nettle [*Urtica dioica* L. (UD)] extract were examined using histopathological and immunohistochemical methods in the stomach tissues of an experimentally created rat model of Crohn’s disease (CD). The CD model was created using trinitrobenzene sulfonic acid (TNBS). The animals in the study were divided into control, TNBS, TNBS+Vit. D, and TNBS+UD groups. At the end of the experiment, the animals were euthanised and their stomach tissues were evaluated for necrosis, degeneration, apoptosis, and inflammation. Additionally, an immunohistochemical method was applied to determine the somatostatin (SSTR), aquaporin-1 (AQP-1), caspase-3, and tumour necrosis factor-alpha (TNF-α) immunoreactivity in the gastric tissues. In the evaluations, degenerative and necrotic changes and mononuclear cell infiltration areas were observed in the TNBS group, but such changes could be improved with Vit. D and UD applications. The results suggest that the combination of the Vit. D and UD extract may have a protective and therapeutic role in mitigating TNBS-induced damage to the gastric tissues, potentially through the regulation of SSTR, AQP-1, caspase-3, and TNF-α expression. This indicates a promising avenue for further research and the exploration of these compounds in the context of gastrointestinal health.

Crohn’s disease (CD) is an inflammatory intestinal disease. It is usually seen in young and middle-aged people and its incidence is about 1** **in 200. The most common symptoms are upper abdominal pain, congestion, and occasional bleeding. Many patients with CD experience serious physical symptoms throughout their lives, such as diarrhoea, vomiting, anorexia, and lethargy, which affect their physical health, quality of life, and mental health (Chuang et al. 2016).

T-cell dysregulation, intestinal dysbiosis, and environmental and dietary factors cause CD in genetically susceptible individuals (Alemany-Cosme et al. 2021). Intestinal tissue damage is known to be influenced by genetic and environmental factors. With the effect of these factors, anomalies occur in the immune response and the mucosa of the intestinal system is affected (Feagan et al. 2016). In CD, treatment planning is undertaken by evaluating the age of the patient, location of symptoms, activity, severity, and complications associated with the disease (Cushing and Higgins 2021).

It has been reported that the risk of colorectal cancer is increased in patients with inflammatory intestine illness compared to the healthy population (Nadeem et al. 2020). The duration of the disease and the anatomical location are direct risk factors in the development of colorectal cancer. Previous research has shown that colorectal cancers developing in patients with inflammatory bowel disease cause higher rates of mortality compared to the normal population (Birch et al. 2022).

Vitamin D_3_ (cholecalciferol) is synthesised from an intermediate metabolite (previtamin D) by the action of ultraviolet-B (UV-B) light (Eliason et al. 2023). Therefore, the levels of vitamin D metabolites in the body change seasonally. Vitamin D deficiency can develop due to a lack of dietary resources or supplements and during the winter months (Janousek et al. 2022).

It has been suggested that vitamin D has a role in the regulation of immune system functions as well as regulating calcium and phosphorus metabolism. It has also been shown that vitamin D deficiency is correlated with the degree of disease activity in CD (Zheng et al. 2023). According to the results of a study investigating the relationship between the incidence of CD and vitamin D deficiency since 2011, vitamin D supplementation has positive effects in the treatment of this disease (White 2018).

The stinging nettle [*Urtica dioica* L. (UD)] is an annual wild-growing herb of the family *Urticaceae*. It has been used in traditional medicine for the treatment of rheumatism and arthritis for centuries. It is also known to have tonic, astringent, and diuretic effects (Bhusal et al. 2022). Because the stinging nettle can increase the level of iron binding, as well as the levels of vitamin B12 and folate in the blood, infusions and the use of the stinging nettle as a detoxifying agent are common in the treatment of anaemia and other disorders (Upton 2013). UD is known as a powerful anti-inflammatory herb and is used as a phytopharmaceutical and food supplement for the treatment of various inflammatory diseases, especially inflammatory intestine diseases (Franciskovic et al. 2017). The carotenoids (e.g., β-carotene, xanthophyll retinoic acid, and retinol), α-tocopherol, ascorbic acid, flavonoids (e.g., kaempferol, quercetin, and rutin), flavone glycosides, catechins, tannins, phenolic compounds (e.g., ferulic, coumaric, and caffeic acid), selenium, and unsaturated fatty acids found in the stinging nettle are components known to be responsible for the antioxidant and anti-inflammatory effects (Zafar et al. 2023).

Somatostatin is a hormone present in many tissues and organs. It is found in the digestive system, nervous system, urinary system, heart, eyes, thymus, thyroid C cells, and pancreatic D cells. It is also abundant in D cells in the antral region of the stomach. D cells are located deep in the stomach and intestinal crypts (Bhanat et al. 2018). Somatostatin (SSTR) has been reported to suppress inflammatory reactions and it is used to treat inflammatory disorders such as psoriasis and rheumatoid arthritis (Mehta and Granstein 2019).

Water is the primary component of all living cells, and the efficient control of water balance is crucial for biological functions to occur. Aquaporins (AQPs), are members of a transmembrane protein family that allow the transport of mainly water and some other small solutes and compounds, such as urea and glycerol, to pass through cell membranes. AQPs are present in almost all living systems and viruses (Ishibashi et al. 2020). AQP-1 has been identified in the gastrointestinal tract from the oesophagus to the colon, and in glial cells and neurons in the enteric nervous system (Volkart et al. 2023). Although the exact role of AQP-1 in the enteric nervous system remains unclear, it has been linked to pathological mechanisms such as pain sensation, inflammatory responses, hypoxic conditions, and diabetes (Wagner et al. 2022).

Caspases are a group of highly conserved cysteine aspartate-specific proteases found in multicellular organisms, and they serve as central controllers of apoptosis. Caspase-3, a member of this family, has been recognised as the principal agent of cell apoptosis. Recent investigations conducted in snails, flies, and rats revealed that caspase-3 serves as a governing factor in synaptic functioning and the generation of neurons (Bishir et al. 2020).

Tumour necrosis factor alpha (TNF-α) is a cytokine, which are cell signalling proteins associated with systemic inflammation, that is among the cytokines responsible for initiating the acute-phase response. TNF can be manufactured by many other cell types, such as lymphocytes, neutrophils, mast cells, eosinophils, NK cells, and neurons, but it is primarily manufactured by active macrophages. The main function of TNF-α is within the realm of immune cells. TNF-α, as an endogenous pyrogen, has the capacity to induce fever, cachexia, apoptotic cell demise, viral replication, and inflammation, and to inhibit tumour formation (Wu et al. 2020).

This study aimed to examine the effects of vitamin D and the stinging nettle on the stomach tissue of rats administered trinitrobenzene sulfonic acid (TNBS).

## MATERIAL AND METHODS

Ethical approval for this study was obtained from the Animal Experiments Local Ethics Committee of Atatürk University’s Faculty of Veterinary Medicine (January 24, 2023, 2023/02). The research was carried out following the principles of the Declaration of Helsinki.

The experimental phase of the study was carried out at the Atatürk University Medical Experimental Research and Application Center using rats procured from the same centre. Thirty-six adult male Sprague-Dawley rats, 3 months old, weighing 200–250 g, were used as the research material. The average weight value of each cage was equalised. The experimental animals used in this study were randomly selected, weighed, and divided into four groups. These groups included the control, TNBS, TNBS+Vit. D, and TNBS+UD groups. After a 24-hour fasting period, the rats were sedated with a dose of 5 mg/kg propofol (Sigma-Aldrich, Istanbul, Türkiye). To induce CD, a single rectal dose of 150 mg/kg TNBS dissolved in physiological saline (Sigma-Aldrich) was administered to all the experimental animals except those in the control group (Zhang et al. 2021). To ensure that the control group rats experienced the same level of stress as the study groups throughout the experiment, a daily application of 1 ml of a physiological saline solution was administered through oral gavage. A single dose of 150 mg/kg TNBS was administered to the TNBS group on the first day of the experiment. On the following days, 1 ml of the physiological saline solution was given by gavage once a day at the same time for 10 days. During the experiment, 7** **500** **IU of vitamin D (Natural Elements, Düsseldorf, Germany) was given to the rats in the TNBS+Vit.** **D group by oral gavage at the same time each day. The rats in the TNBS+UD group were given a 2.5 ml/kg UD extract by oral gavage at the same time each day throughout the experiment. At the end of the experiment, deep sedation of the rats was achieved with sevoflurane (Sevorane^®^, Abbott Laboratories, Istanbul, Türkiye) and cervical dislocation was performed. Stomach tissue specimens were collected and immersed in a 10% buffered neutral formalin solution. Following standard histological protocols, they were subsequently embedded in paraffin.

### Plant extract

The leaves of the UD plants were dried in an incubator at 40 °C. After the dried plant samples were pulverised, 250 g of the dried plant was taken and extracted in a polar solvent (water) using the Soxhlet extraction method. The solvent was evaporated with the help of a rotary evaporator and lyophilised. A stock solution was prepared at a concentration of 25 mg/ml from the lyophilised plant extract and used in serial dilutions (DMSO was used at > 1/1 000 as solvent).

### Histopathological examinations

Sections of 5 μm taken from paraffin blocks and mounted on slides were stained with haematoxylin and eosin (H&E) and evaluated under a light microscope (Nikon BX51) in terms of the necrotic and degenerative changes as follows: none (0), mild (1), moderate (2), or severe (3) (Gezer and Karadag-Sari 2022).

### Immunohistochemical examinations

Endogenous peroxidase inactivation was achieved by keeping the 5 μm sections on poly l-lysine slides after passing them through xylol and alcohol series and washing them with phosphate buffered saline (PBS) for 10 min in 3% H_2_O_2_. To reveal the antigens in the tissues, they were treated with an antigen retrieval solution for 2 × 5 min at 500 W. Subsequently, the tissues were incubated overnight with SSTR (Abcam; Cat. No. ab183855), AQP-1 (Abcam; Cat. No. ab9566), caspase-3 (Biorbyt, Cat. No. orb382909), and TNF-α (Santa Cruz; Cat. No. sc-133192) primary antibodies (dilution: 1/200). Additionally, a high-capacity detection system was employed utilising Anti-Polyvalent HRP (Thermo Fisher; Cat. No. TP-125-HL), following the manufacturer’s instructions. DAB (3,3'-diaminobenzidine) was used as the chromogen. Subsequently, specimens were counterstained with Mayer’s haematoxylin, covered with Entellan, and observed under a light microscope.

Evaluations of the immunoreactivity were performed semi-quantitatively as follows: none (0), mild (1), moderate (2), or severe (3) (Gezer et al. 2023).

### Statistical analysis

The data were analysed with IBM SPSS Statistics v20.00. The normality of the data was evaluated with the Kolmogorov-Smirnov test. Differences between the groups were determined with the Kruskal-Wallis test. The Mann-Whitney *U* test was used for the pairwise comparisons (*P <* 0.05).

## RESULTS

### Histopathological results

Statistically significant differences were found between the groups in the histopathological examinations (Table 1, *P* *<* 0.05).

**Table 1:** Comparison of the groups in terms of mucosal injuries, mononuclear cell infiltration, SSTR, AQP-1, caspase-3, and TNF-α levels

Groups	Gastric mucosa injury	Mononuclear cell infiltrations	SSTR	AQP-1	Caspase-3	TNF-α
Control	0.16 ± 0.40^a^	0.00 ± 0.00^a^	2.83 ± 0.40^a^	2.00 ± 0.00^a^	0.16 ± 0.40^a^	0.16 ± 0.40^a^
TNBS	2.83 ± 0.40^b^	2.16 ± 0.40^b^	2.00 ± 0.00^b^	1.16 ± 0.40^b^	2.66 ± 0.51^b^	2.83 ± 0.40^b^
TNBS+Vit. D	1.16 ± 0.40^c^	1.00 ± 0.40^c^	2.66 ± 0.51^a^	2.16 ± 0.40^a^	1.16 ± 0.40^c^	1.33 ± 0.51^c^
TNBS+UD	1.33 ± 0.51^c^	1.16 ± 0.40^c^	2.83 ± 0.40^a^	1.83 ± 0.40^a^	1.00 ± 0.00^c^	1.00 ± 0.00^c^

^a–c^Show differences between groups (*P *< 0.05)

AQP-1 = aquaporin-1; SSTR = somatostatin; TNBS = trinitrobenzene sulfonic acid; TNF-α = tumour necrosis factor-alpha; UD = *Urtica dioica*; Vit. D = Vitamin D_3_

**Figure 1:**
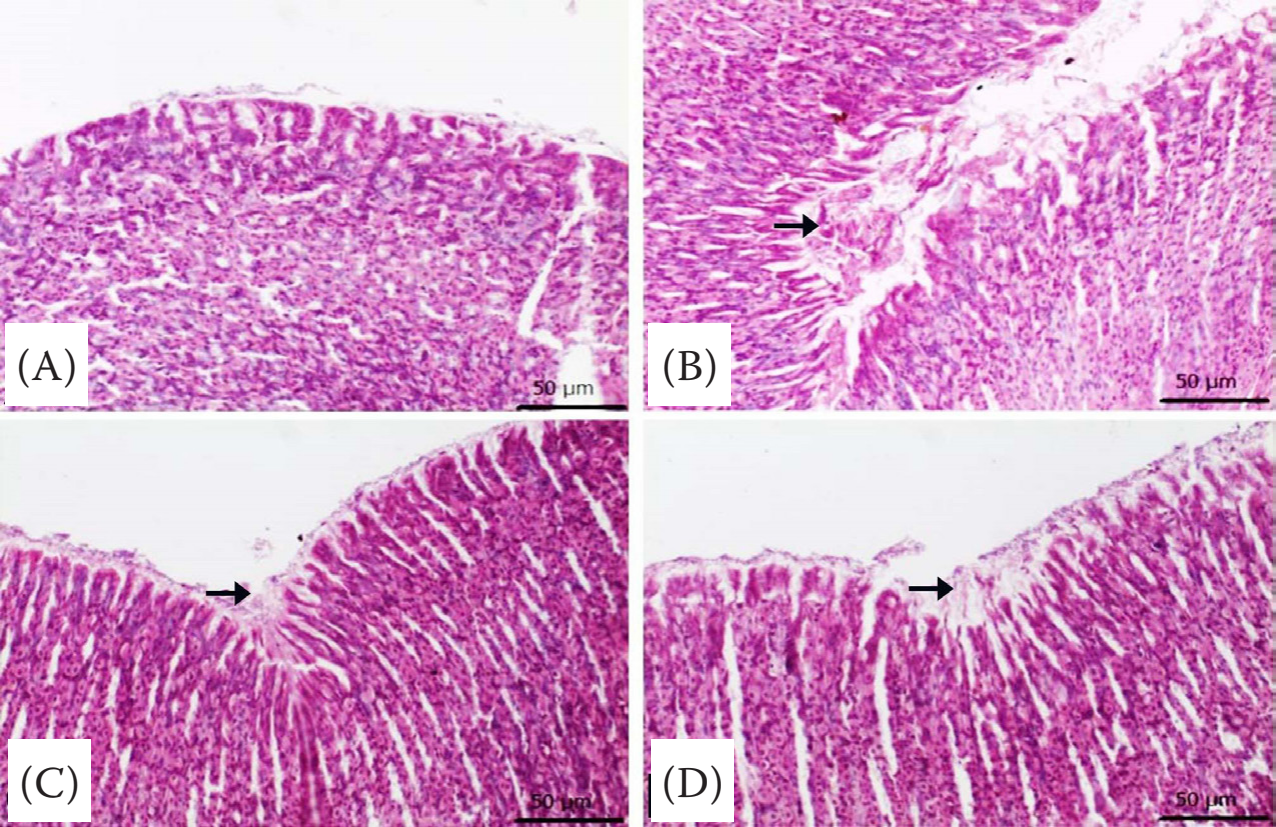
Histological changes in rat stoma tissue (A) Control group; stomach tissue. Normal view. (B) TNBS group; severely degenerative areas of the mucosal epithelium (arrow). (C) TNBS+Vit. D group; mildly degenerative areas of the mucosal epithelium (arrow). (D) TNBS+UD group; mildly degenerative areas of the mucosal epithelium (arrow). ×** **50 (H&E) H&E = haematoxylin&eosin; TNBS = trinitrobenzene sulfonic acid; UD = *Urtica dioica;* Vit. D = Vitamin D_3_

**Figure 2:**
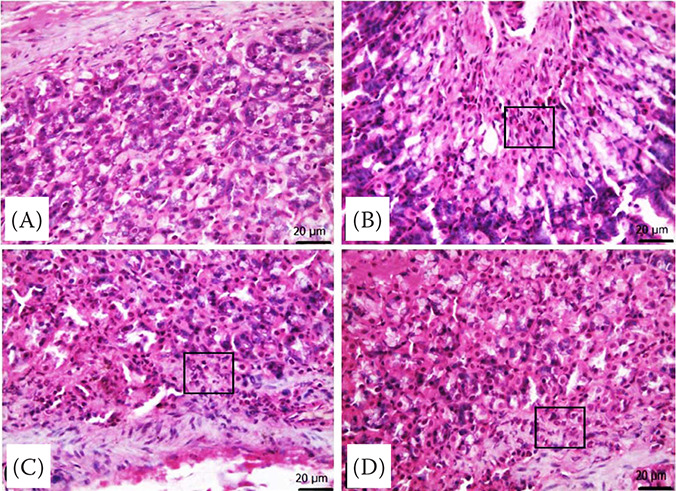
Histological changes in rat stoma tissue (A) Control group; stomach tissue. Normal view. (B) TNBS group; moderate mononuclear cell infiltrates near the submucosa (□). (C) TNBS+Vit. D group; mild mononuclear cell infiltrates (□). (D) TNBS+UD group; mild mononuclear cell infiltrates (□). × 20 (H&E) H&E = haematoxylin&eosin; TNBS = trinitrobenzene sulfonic acid; UD = *Urtica dioica;* Vit. D = Vitamin D_3_

The stomach tissue of the rats in the control group had a normal histological appearance. While severe degenerative and necrotic changes were observed in the mucosal epithelium of the TNBS group, mild degenerative and necrotic changes were observed in the groups given vitamin D and UD together with TNBS. In addition, it was determined that mononuclear cell infiltrations, which were seen at a moderate level in the submucosal areas of the TNBS group, were decreased to a mild level in the vitamin D and UD groups (Figures 1 and 2).

### Immunohistochemical results

Statistically significant differences were found between the groups in the immunohistochemical examinations (Table 1, *P* *<* 0.05).

**Figure 3:**
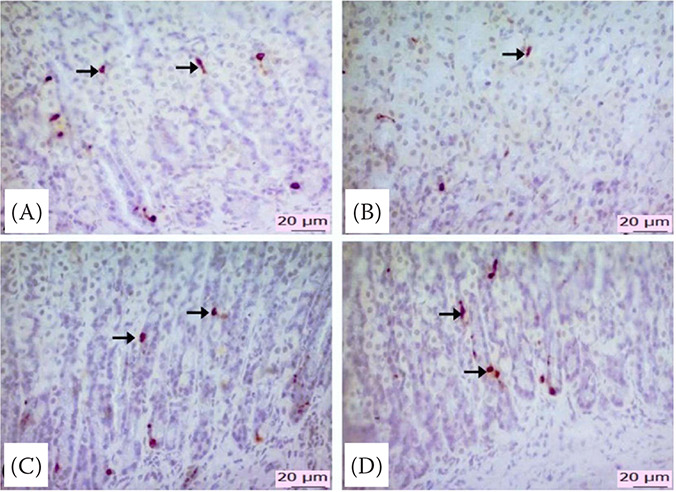
Immunopositivity of somatostatin (A) Control group; severe immunopositivity. (B) TNBS group; moderate immunopositivity. (C) TNBS+Vit. D group; severe immunopositivity. (D) TNBS+UD group; severe immunopositivity (arrows). ×** **20 (IHC) IHC = intro to immunohistochemistry; TNBS = trinitrobenzene sulfonic acid; UD = *Urtica dioica;* Vit. D = Vitamin D_3_

**Figure 4:**
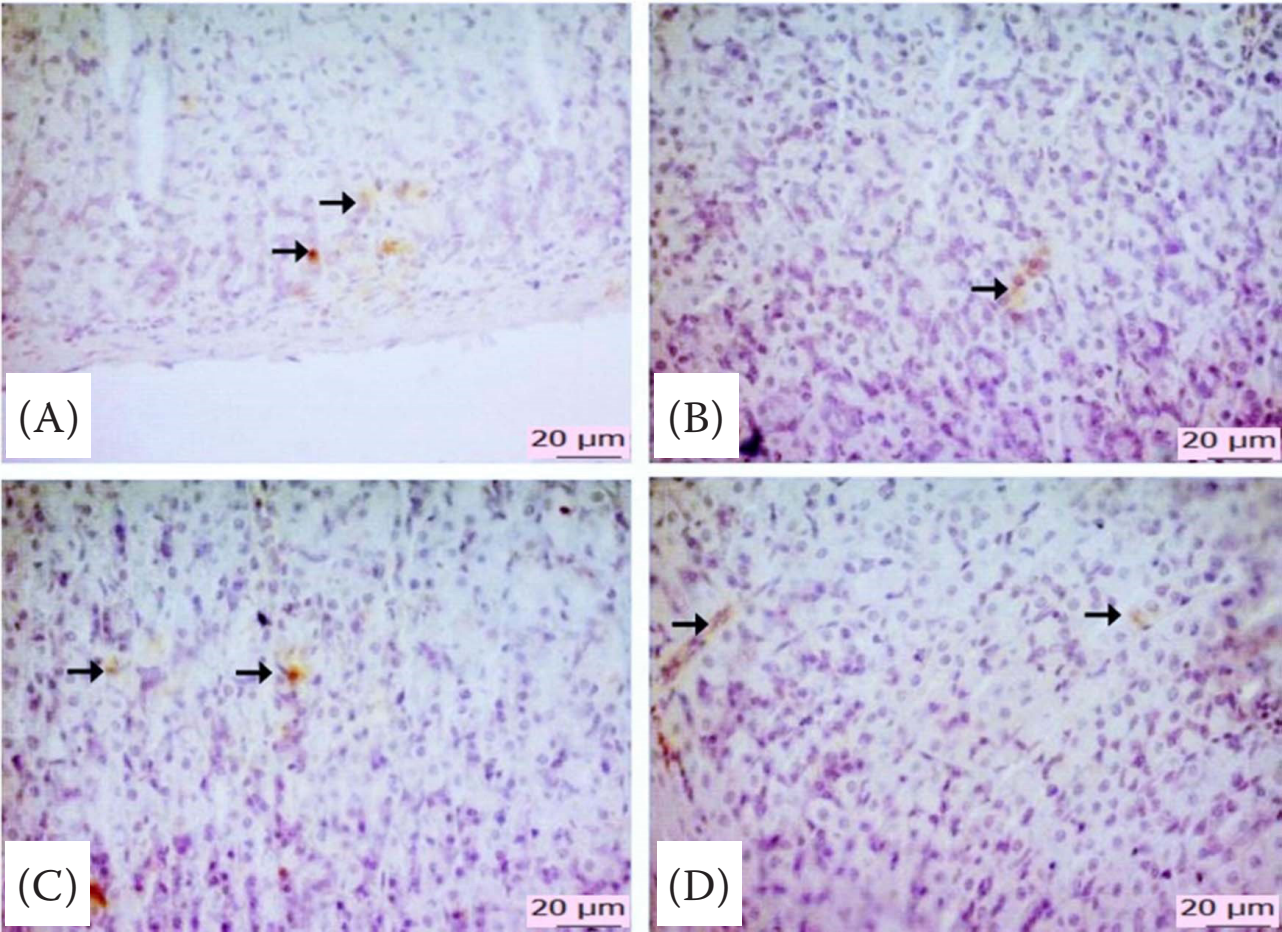
Immunopositivity of aquaporin-1 (A) Control group; moderate immunopositivity. (B) TNBS group; mild immunopositivity. (C) TNBS+Vit. D group; moderate immunopositivity. (D) TNBS+UD group; moderate immunopositivity (arrows). × 20 (IHC) IHC = intro to immunohistochemistry; TNBS = trinitrobenzene sulfonic acid; UD = *Urtica dioica;* Vit. D = Vitamin D_3_

As a result of the immunohistochemical staining with SSTR, it was determined that the immunopositivity was severe in the control, TNBS+Vit. D, and TNBS+UD groups, whereas it was moderate in the group treated with TNBS alone. The immunopositivity results for aquaporin were similar to those of SSTR. AQP-1 immunopositivity, which was moderate in the control, TNBS+Vit. D, and TNBS+UD groups, was mild in the TNBS group (Figures 3 and 4).

**Figure 5:**
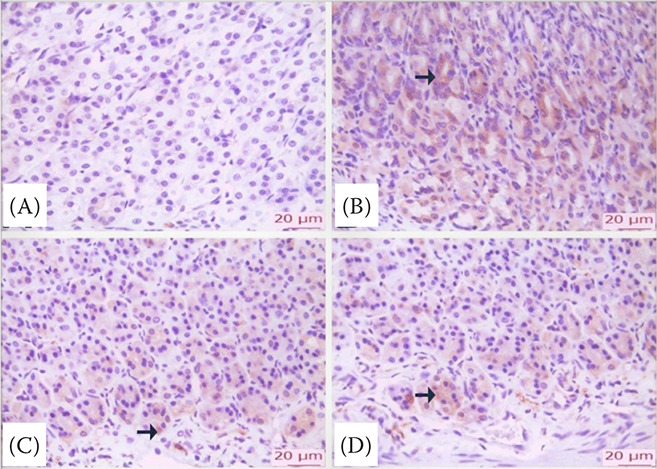
Immunopositivity of caspase-3 (A) Control group; immunonegativity. (B) TNBS group; severe immunopositivity. (C) TNBS+Vit D group; mild immunopositivity. (D) TNBS+UD group; mild immunopositivity (arrows). ×** **20 (IHC) IHC = intro to immunohistochemistry; TNBS = trinitrobenzene sulfonic acid; UD = *Urtica dioica;* Vit. D = Vitamin D_3_

**Figure 6:**
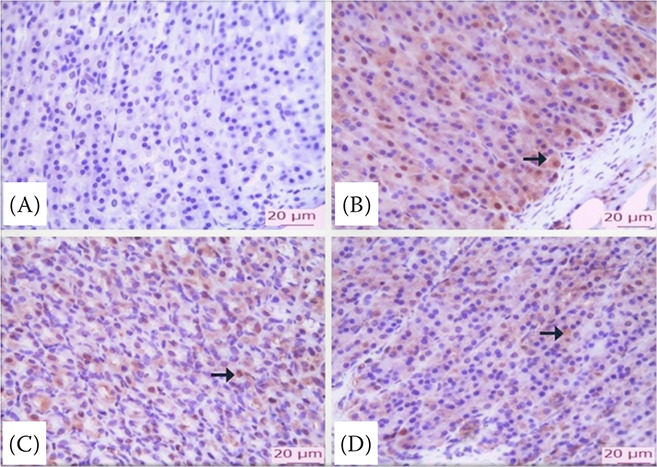
Immunopositivity of TNF-α (A) Control group; immunonegativity. (B) TNBS group; severe immunopositivity. (C) TNBS+Vit. D group; moderate immunopositivity. (D) TNBS+UD group; moderate immunopositivity (arrows). ×** **20 (IHC) IHC = intro to immunohistochemistry; TNBS = trinitrobenzene sulfonic acid; UD = *Urtica dioica;* Vit. D = Vitamin D_3_

The staining for caspase-3 and TNF-α did not show significant immunopositivity in the control group. On the other hand, the levels of caspase-3 and TNF-α immunopositivity were both severe in the TNBS group. The immunopositivity of caspase-3 was decreased to a mild level and the immunopositivity of TNF-α was decreased to a moderate level in the TNBS+Vit. D and TNBS+UD groups (Figures 5 and 6).

## DISCUSSION

CD usually arises from the proximal jejunum and often spreads rapidly to the duodenum and stomach. In addition to apoptosis and inflammation, excessive immune response to various antigens or environmental factors plays a role in the aetiology of this disease (Terra et al. 2021). It is rare for CD to not affect the stomach and duodenum, and the appearance of granulomas is very obvious and easy to identify. Focal acute inflammation of the gastroduodenum, especially in the non-inflamed mucosal background, is known to be a typical clinical manifestation of CD. However, proper and careful sampling from the stomach and duodenum is required (Rogler et al. 2021). In a comprehensive review, it was stated that the most common histopathological finding of CD is non-specific gastric inflammation.

Additionally, gastric granulomas can also be observed and focal gastritis is common. Gastric inflammation, duodenal inflammation, and gastric granuloma are seen in patients with CD in the upper intestinal tract (Abuquteish and Putra 2019). In the present study, moderate degenerative changes and mononuclear cell infiltrations in the submucosal areas were observed in the TNBS group, while these were mild in the vitamin D and UD groups. Based on these findings, it is thought that vitamin D and UD may have positive effects on the recovery of patients with CD.

It has been shown in various studies that an insufficient amount of vitamin D may play a role in the pathogenesis of CD, and administration of vitamin D is considered promising in the treatment of CD. In addition, vitamin D supplementation is safe, inexpensive, and readily available, making its potential therapeutic applications all the more attractive (Wu et al. 2022). In light of this information from the literature and considering that vitamin D has a positive effect on the course of CD, this study was planned and the therapeutic effect of vitamin D in the event of damage caused by TNBS was investigated.

In the traditional medical treatment of CD, the aimed is to reduce the level of TNF-α, as a high-level inflammatory protein, with anti-TNF drugs. However, the long-term administration of anti-TNF drugs may decrease in effectiveness over time. It has been reported that a UD leaf extract plays a positive role in the treatment of inflammatory illness by effectively suppressing cytokines (Dhouibi et al. 2020). UD extracts have been shown to be effective in reducing inflammation that causes colitis (Bhusal et al. 2022). Genç et al. used a UD extract in the treatment of experimentally induced colitis in rats. The values of TNF-α cytokines and IL-1β were decreased in the faeces of rats administered UD, once again reflecting an improvement in the symptoms of CD (Genc et al. 2011). Kikut et al. (2021) reported that UD played a positive role in the treatment of inflammatory illness. In the present study, which was planned in light of the aforementioned literature findings, it was shown both histopathologically and immunohistochemically that the UD extract had healing effects on gastric tissue in an experimental model of CD.

Somatostatin is an endogenous hormone that has the effects of decreasing enteric secretion and motility, boosting water and electrolyte absorption, and inhibiting pancreatic exocrine secretion. Some studies have demonstrated that SSTR or its analogues can be used for the treatment of gastrointestinal inflammation diseases (Gadelha et al. 2022). Inflammatory cytokines also showed regulatory effects against SSTR receptor secretion. While SSTR secretion is stimulated by IL-1, IL-6, and IL-10, it is inhibited by TGF-β. Therefore, SSTR secretion is stimulated in cases of inflammatory diseases such as CD. SSTR secretion increases in treated patients (Caruso et al. 2018). In the present study, results similar to those of previous studies in the literature were obtained. The somatostatin levels determined by the immunohistochemistry method were found to be higher in the groups given vitamin D and stinging nettle.

AQPs are a family of proteins that selectively and efficiently transport water and/or other minor uncharged solutes across biological membranes. Studies of AQPs have provided important insights into the mechanisms that mediate water homeostasis in human disease and health (Wagner et al. 2022). These proteins play a significant role in bowel function via fluid homeostasis. In a previous experimental CD model created with TNBS and dextran sodium sulfate, it was shown that the level of AQP-1 was decreased in cases of CD (Gao et al. 2020). In a study of inflammatory intestinal disease with characteristics similar to CD, it was reported that the AQP levels were decreased due to inflammation (Meli et al. 2018). In our study, we obtained results similar to those of these previous studies in the literature. In our study, the AQP-1 value determined by the immunohistochemistry method was mild in the TNBS group, while it was moderate in the TNBS+Vit. D and TNBS+UD groups. These results show that vitamin D and UD extracts may have positive roles in the treatment of CD.

Proinflammatory cytokines, such as IL-1β and TNF-α, have key roles in inflammation-related diseases. Decreases in the levels of these inflammatory cytokines play important roles in the treatment of diseases (Wang et al. 2020). In this study, it was determined that the level of the mononuclear cell infiltration detected by the histopathology method was higher in the TNBS group, but it decreased in the treatment groups. Thus, it can be suggested that vitamin D and UD have positive roles in reducing inflammatory cytokines. In a study conducted to consider the immunomodulatory properties of vitamin D, it was found that the TNF-α levels were normal and the IL-10 levels were low in CD patients with vitamin D deficiency. Thus, a relationship between inflammatory diseases and vitamin D deficiency was revealed (Ao et al. 2021). In this study, in accordance with the literature, it was determined that the inflammation in the groups given vitamin D or UD was reduced compared to the TNBS group. The decrease in the TNF-α levels according to the immunohistochemical method supported this finding. Accordingly, it was revealed that vitamin D and UD had positive effects in the treatment of CD.

Abnormal levels of caspase, which programmes and is responsible for apoptosis, is a risk factor for CD. Cell division by caspase-3 under stress may play a role in the expression of CD. Therefore, a reduction in the caspase enzyme is important in cases of CD (Kopiasz et al. 2021). Experimental CD studies have revealed that the apoptotic activation is higher in CD groups compared to controls (Lykowska-Szuber et al. 2021).

Decreases in the levels of caspase and TNF-α, which are responsible for apoptosis, may play an effective role in the treatment mechanism of CD (Lykowska-Szuber et al. 2023). In this study, similar to the findings of other studies, it was determined that caspase levels were lower in the groups receiving vitamin D and UD compared to the TNBS group. Accordingly, it was determined that vitamin D and UD had positive effects on CD.

CD is known today as one of the most common diseases related to diseases of the digestive system. The treatment of this disease is costly and time-consuming, and the last resort is surgical treatment. Therefore, research, such as that presented here, is of great importance in order to offer both preventive and therapeutic alternatives to patients suffering from this disease.
